# Full-thickness closure using reopenable clip-over-the-line method for a large defect after duodenal full-thickness resection

**DOI:** 10.1055/a-2590-7986

**Published:** 2025-05-22

**Authors:** Tatsuma Nomura, Morihito Setsuda, Shinichiro Nakamura, Takashi Hamada, Hiroshi Kaneko, Katsumi Mukai

**Affiliations:** 1 Department of Gastroenterology, Suzuka General Hospital, Suzuka, Mie, Japan; 2 Department of Endoscopy Center, Suzuka General Hospital, Suzuka, Mie, Japan; 3 Department of Surgery, Suzuka General Hospital, Suzuka, Mie, Japan

Laparoscopic-assisted full-thickness resection of duodenal gastrointestinal stromal tumors (GISTs) is a safe and reliable procedure 1 . However, due to the narrow lumen of the duodenum, there exists a possibility that the mucosal closure on the gastrointestinal side could be insufficient 2 . We have previously described the feasibility of the reopenable clip-over-the-line method (ROLM), which can be used to close large mucosal defects even in narrow lumens 3 4 . In this report, we present a case of full-thickness defect closure of the anterior wall of the duodenal bulb using the ROLM.

A male patient in his 60s was diagnosed with a GIST by biopsy of the anterior wall of the duodenal bulb ( Fig. 1 , Video 1 ). We completely resected the GIST laparoscopically. The bulb defect was approximately 25 mm in size. We used the ROLM to close the defect. First, a clip with a line was placed to grasp the edge of the defect on the anal side, including the serous muscle layer. A reopenable clip with a line threaded through one of the teeth placed on the contralateral edge to grasp the mucosa and fat tissue. Even in a narrow lumen, such as the duodenum, the use of a tapered tip hood made it possible to repeatedly place the reopenable-clips without existing clips obstructing the view 5 . Laparoscopic observation confirmed that the defect was completely closed, except for the defect near the pyloric ring, and the serous muscle layer was surgically sutured. The pyloric stenosis was confirmed to be free from narrowing under endoscopic visualization, and the remaining full-thickness defect was completely closed. The patient was discharged from the hospital 7 days postoperatively without any adverse events. Five weeks later, endoscopy confirmed that the clips were still in place and that there was no gastrointestinal stenosis.

Endoscopy_UCTN_Code_TTT_1AO_2AZ

**Fig. 1:**
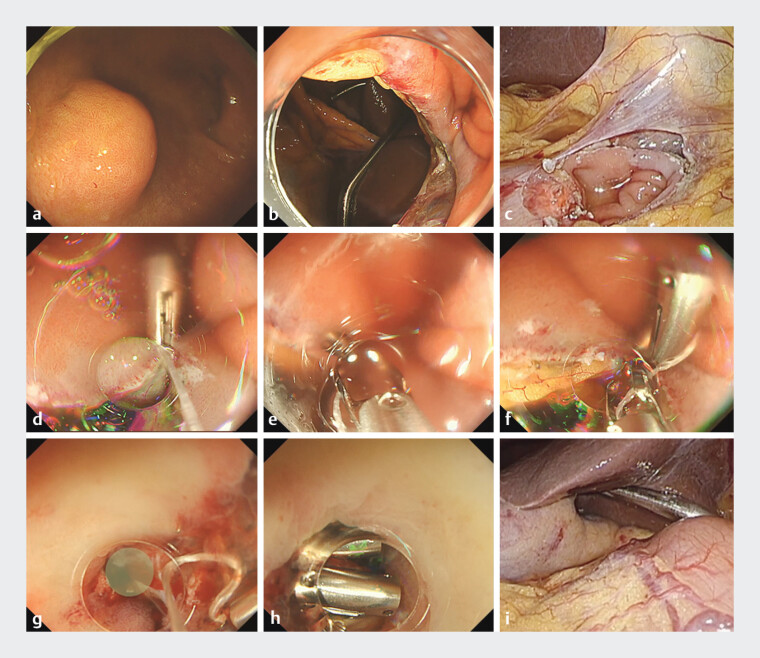
Closure of a large full-thickness defect in the duodenum using the reopenable clip-over-the-line method (ROLM). **a** A 13-mm gastrointestinal stromal tumor (GIST) in the anterior wall of the duodenal bulb. **b** Full-thickness defect of the anterior duodenal bulb up to the pyloric ring, approximately 25 mm in size, after full-thickness resection of the SMT. **c** Full-thickness defect of the duodenum observed via laparoscopy. **d** The first clip with a line is placed on the anal side of the duodenum. **e** A clip with a line threaded through a tooth is placed on the opposite side to grasp the mucosa and serous muscle layers. **f** If fat tissue is identified, the clip is placed by grasping the fat in addition to the mucosa and serous muscle layers. **g** A small full-thickness defect (green area) is identified near the pyloric ring after surgical serous suturing. **h** After confirming the absence of stenosis of the pyloric ring under endoscopic visualization, the remaining full-thickness defect is completely closed. **i** After observing the duodenum in the abdominal cavity, the absence of gas leakage due to insufflation through the endoscope is confirmed. Abbreviations: GIST gastrointestinal stromal tumors; ROLM, reopenable clip-over-the-line method.

Closure of a large full-thickness defect in the duodenum using the reopenable clip-over-the-line method.Video 1

## Notes

Endoscopy E-Videos https://eref.thieme.de/e-videos
*E-Videos* is an open access online section of the journal *Endoscopy* , reporting on interesting cases and new techniques in gastroenterological endoscopy. All papers include a high-quality video and are published with a Creative Commons CC-BY license. Endoscopy E-Videos qualify for HINARI discounts and waivers and eligibility is automatically checked during the submission process. We grant 100% waivers to articles whose corresponding authors are based in Group A countries and 50% waivers to those who are based in Group B countries as classified by Research4Life (see: https://www.research4life.org/access/eligibility/ ). This section has its own submission website at https://mc.manuscriptcentral.com/e-videos .
